# Positive Interactions between Lactic Acid Bacteria Promoted by Nitrogen-Based Nutritional Dependencies

**DOI:** 10.1128/AEM.01055-21

**Published:** 2021-09-28

**Authors:** Fanny Canon, Marie-Bernadette Maillard, Gwénaële Henry, Anne Thierry, Valérie Gagnaire

**Affiliations:** a UMR STLO, INRAE, Institut Agro, Rennes, France; University of Helsinki

**Keywords:** lactic acid bacteria, positive interactions, commensalism, nutritional dependency, nitrogen nutrition, functional outputs

## Abstract

Nutritional dependencies, especially those regarding nitrogen sources, govern numerous microbial positive interactions. As for lactic acid bacteria (LAB), responsible for the sanitary, organoleptic, and health properties of most fermented products, such positive interactions have previously been studied between yogurt bacteria. However, they have never been exploited to create artificial cocultures of LAB that would not necessarily coexist naturally, i.e., from different origins. The objective of this study was to promote LAB positive interactions, based on nitrogen dependencies in cocultures, and to investigate how these interactions affect some functional outputs, e.g., acidification rates, carbohydrate consumption, and volatile-compound production. The strategy was to exploit both proteolytic activities and amino acid auxotrophies of LAB. A chemically defined medium was thus developed to specifically allow the growth of six strains used, three proteolytic and three nonproteolytic. Each of the proteolytic strains, Enterococcus faecalis CIRM-BIA2412, Lactococcus lactis NCDO2125, and CIRM-BIA244, was cocultured with each one of the nonproteolytic LAB strains, L. lactis NCDO2111 and Lactiplantibacillus plantarum CIRM-BIA465 and CIRM-BIA1524. Bacterial growth was monitored using compartmented chambers to compare growth in mono- and cocultures. Acidification, carbohydrate consumption, and volatile-compound production were evaluated in direct cocultures. Each proteolytic strain induced different types of interactions: strongly positive interactions, weakly positive interactions, and no interactions were seen with E. faecalis CIRM-BIA2412, L. lactis NCDO2125, and L. lactis CIRM-BIA244, respectively. Strong interactions were associated with higher concentrations of tryptophan, valine, phenylalanine, leucine, isoleucine, and peptides. They led to higher acidification rates, lower pH, higher raffinose utilization, and higher concentrations of five volatile compounds.

**IMPORTANCE** Interactions of lactic acid bacteria (LAB) are often studied in association with yeasts or propionibacteria in various fermented food products, and the mechanisms underlying their interactions are being quite well characterized. Concerning interactions between LAB, they have mainly been investigated to test antagonistic interactions. Understanding how they can positively interact could be useful in multiple food-related fields: production of fermented food products with enhanced functional properties or fermentation of new food matrices. This study investigated the exploitation of the proteolytic activity of LAB strains to promote positive interactions between proteolytic and nonproteolytic strains. The results suggest that proteolytic LAB do not equally stimulate nonproteolytic LAB and that the stronger the interactions between LAB are, the more functional outputs we can expect. Thus, this study gives insight into how to create new associations of LAB strains and to guarantee their positive interactions.

## INTRODUCTION

Lactic acid bacteria (LAB) are the most prevalent bacterial actors in fermented foods consumed in Western countries (1). LAB can produce a variety of compounds, including weak organic acids, e.g., lactic and acetic acids, aroma compounds such as diacetyl, amino acids, peptides, exopolysaccharides, and vitamins, as well as hydrolytic enzymes, hydrogen peroxide, and bacteriocins, during fermentations (2). These compounds provide fermented foods with varied desirable functional outputs, such as organoleptic, sanitary, nutritional, probiotic, and health properties (3–6). However, their production in LAB-fermented foods is both species and strain dependent, and consequently, the “superstrain” that would produce all the expected metabolites does not exist (7). To increase the functional outputs, we need to find efficient ways to associate strains with complementary properties in an artificial coculture, defined as an association of microorganisms that may not necessarily be found in nature (8). Coculture can also increase substrate conversion, yields, and microbial fitness, in particular when microorganisms interact positively with each other, through either commensalism, cooperation, mutualism, or syntrophy (8).

The exploitation of nutritional dependencies seems to be one of the most promising ways for LAB to interact in coculture. The exchange of nitrogen compounds either as public goods, defined as the pool of molecules available in the medium (9), or through cross-feeding, i.e., the phenomenon by which one microorganism takes in a primary substrate and converts it into a product excreted as a public good (10), is of particular interest. For example, dependencies based on nitrogen nutrients have been observed between the yeast Saccharomyces cerevisiae and the LAB Lactococcus lactis (11), as well as between the two LAB species, used as a prime example, Streptococcus salivarius subsp. *thermophilus* and Lactobacillus delbrueckii subsp. *bulgaricus*, associated in yogurt (12). In milk fermentation, the sharing of extracellular protease activity, especially by LAB species such as L. lactis, is paramount to ensure microbial interactions in cheese and fermented milks (13). Protease activity is unevenly distributed among the strains, rendering the growth of the strains that lack this activity dependent on the composition of the medium in peptides and free amino acids.

LAB cocultures were recently shown to efficiently ferment mixes of plant-based substrates and milk (14, 15) to meet the growing need of new substrate use in the context of food transition. Until now, most LAB strains were mainly selected from dairy applications, but we need to find more suitable candidates to ferment plant-sourced substrates as well. Efficient cocultures of LAB strains adapted to both dairy and plant-based resources have been achieved by our team in a previous work in which functional complementarity of selected LAB strains was exploited to design cocultures able to ferment mixes of milk and lupin (15). More specifically, we associated nonproteolytic strains able to degrade specific carbohydrates with proteolytic strains, the latter being expected to provide the former with nitrogen nutrients. As the culture medium was initially rich in nitrogen compounds, and four to six strains were associated in coculture, this complicated the study of the nitrogen-related interactions between the LAB.

**FIG 1:**
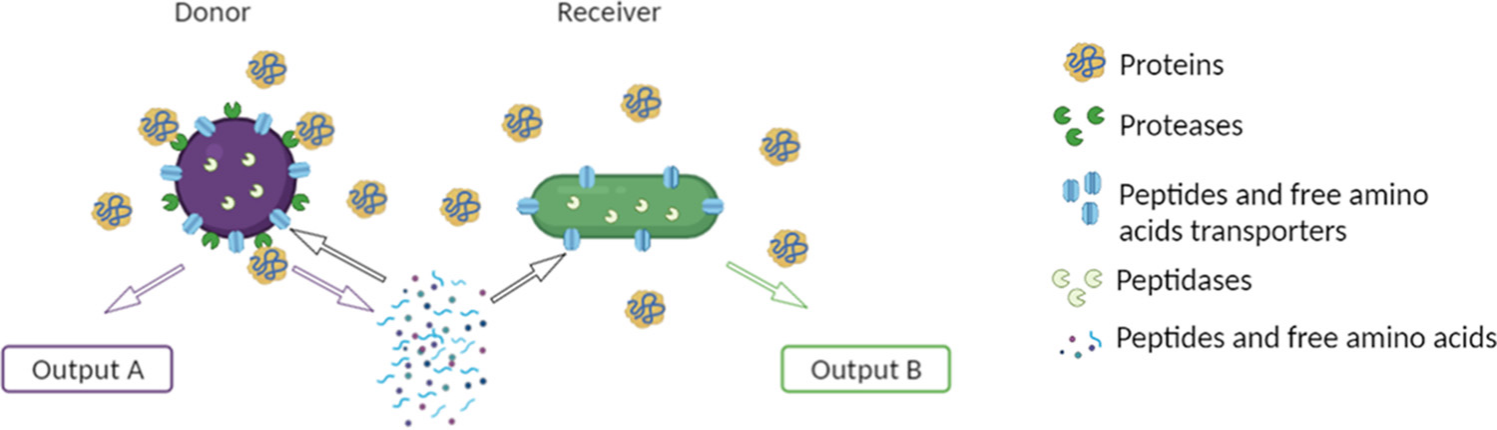
Schematic representation of the strategy used to favor positive interactions between two strains of lactic acid bacteria. The first strain is selected for its proteolytic activity and its capacity to produce output A (e.g., aroma compounds). Its protease(s) will degrade the proteins present in the medium into peptides and free amino acids. The first strain is hence referred to as the donor. The second strain is selected for its capacity to produce output B (e.g., carbohydrate hydrolysis), its lack of proteolytic capacity, and its amino acid auxotrophies. It will then benefit from the pool of peptides and free amino acids available and is hence referred to as the receiver. The objective is to favor commensalism or mutualism between the two strains in coculture.

The objective of this study was to promote LAB positive interactions based on nitrogen dependencies in artificial cocultures and to investigate the possible functional outputs of such interactions. For this, LAB strains were deliberately associated in a model medium mimicking a mix of plant-based substrates and milk. We selected two types of strains from various dairy and nondairy/vegetable origins. The first ones, referred to as donor strains, were selected for their proteolytic activity and their contribution to the development of flavor. Lactococcus lactis and Enterococcus faecalis were found to be adequate candidates. Both species possess a very efficient proteolytic system and can modify sensorial profiles in cheeses and other fermented dairy products (16, 17). The E. faecalis proteolytic system was even shown to be responsible for a decrease of allergenicity of bovine milk proteins (18). The second type of strains, referred to as receiver strains, were selected, in contrast, for their lack of proteolytic activity and their ability to consume raffinose family oligosaccharides (RFO), which are responsible for intestinal discomfort. Strains of L. lactis and Lactiplantibacillus plantarum that were previously shown to exhibit this capacity (15) were selected for the present study (Fig. 1).

In the present study, we first developed a chemically defined medium that contained all necessary vitamins, minerals, and nucleic acids, as well as the proteins and carbohydrates of bovine milk and lupin. Second, we associated a donor strain with a receiver one. We used either compartmented chambers or direct cocultures to study LAB interactions and compared them to monocultures. The present study demonstrated that it is possible to promote positive interactions based on nutritional dependencies between LAB strains by exploiting both their proteolytic activities and their amino acid auxotrophies. Regarding the functional outputs that result from LAB interactions, we investigated in particular the acidification rates to ensure safety, RFO consumption to reduce intestinal discomfort, and production of aroma compounds to improve sensory properties. The results indicate that different proteolytic LAB do not equally stimulate nonproteolytic LAB. They further suggest that the stronger the interactions between LAB are, the more functional outputs we can expect.

## RESULTS

### Selection of the proteolytic (donor) and nonproteolytic (receiver) strains and validation of a chemically defined medium to study LAB interactions.

**FIG 2:**
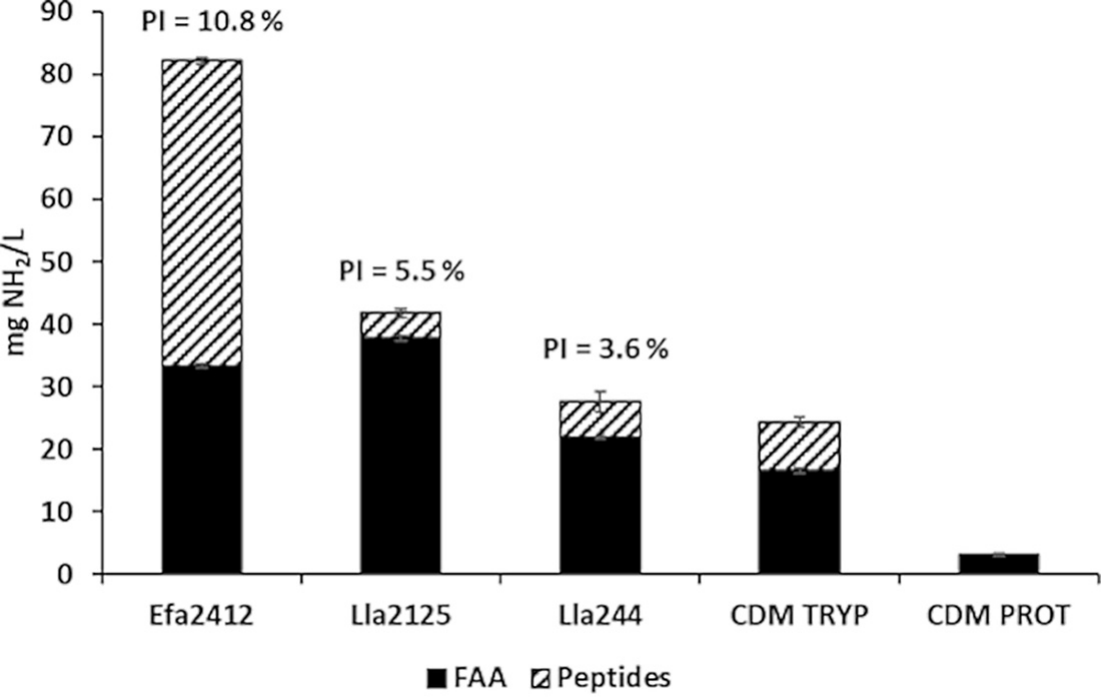
Donor strains were selected for their distinct proteolytic profiles. Concentrations of free NH_2_ groups after 24 h of fermentation of CDM PROT by donor strains Efa2412, Lla2125, and Lla244 and those present in CDM TRYP and CDM PROT prior to culture. The proteolytic index (PI) obtained for each donor strain cultured in CDM PROT was calculated as the ratio of the peptides and free amino acids (FAA) measurable by OPA during fermentation (OPA_sample_) and the OPA_max_. The total amount of free NH_2_ has been separated into the part related to free amino acids and the one related to peptides, calculated by difference between total and free amino acid-related NH_2_ groups, converted in OPA response using the free amino acid dosage. For strain codes, see Table 4.

The donor strains were selected according to their proteolytic profiles, i.e., the NH_2_-containing compounds they provided, when grown in the chemically defined medium that contained casein and/or lupin proteins as sole nitrogen source (CDM PROT). The concentrations of free amino acids (FAA) and peptides in the medium after fermentation by each of the donor strains are illustrated in Fig. 2. FAA and peptides resulted from the hydrolysis of casein and/or lupin proteins present in the CDM PROT. E. faecalis CIRM-BIA2412 (Efa2412) appeared as the most proteolytic strain, with proteolytic indices (PI) of 10.8% ± 0.1% versus 5.5% ± 0.1% and 3.6% ± 0.3% for L. lactis NCDO2125 (Lla2125) and L. lactis CIRM-BIA244 (Lla244), respectively.

The concentration of total FAA in CDM PROT increased from 4.8 mg/liter initially to 250, 261, and 155 mg/liter after fermentation by Efa2412, Lla2125, and Lla244, respectively. The concentration of peptides released by Efa2412 (48.8 mg NH_2_/liter) was significantly higher than those released by the two other donor strains, Lla244 (5.8 mg NH_2_/liter) and Lla2125 (4.0 mg NH_2_/liter). Among all the free NH_2_ groups available in medium after the fermentation by Efa2412, Lla2125 and Lla244, peptides represented 59.4%, 9.6%, and 20.7%, for each donor strain, respectively.

**FIG 3:**
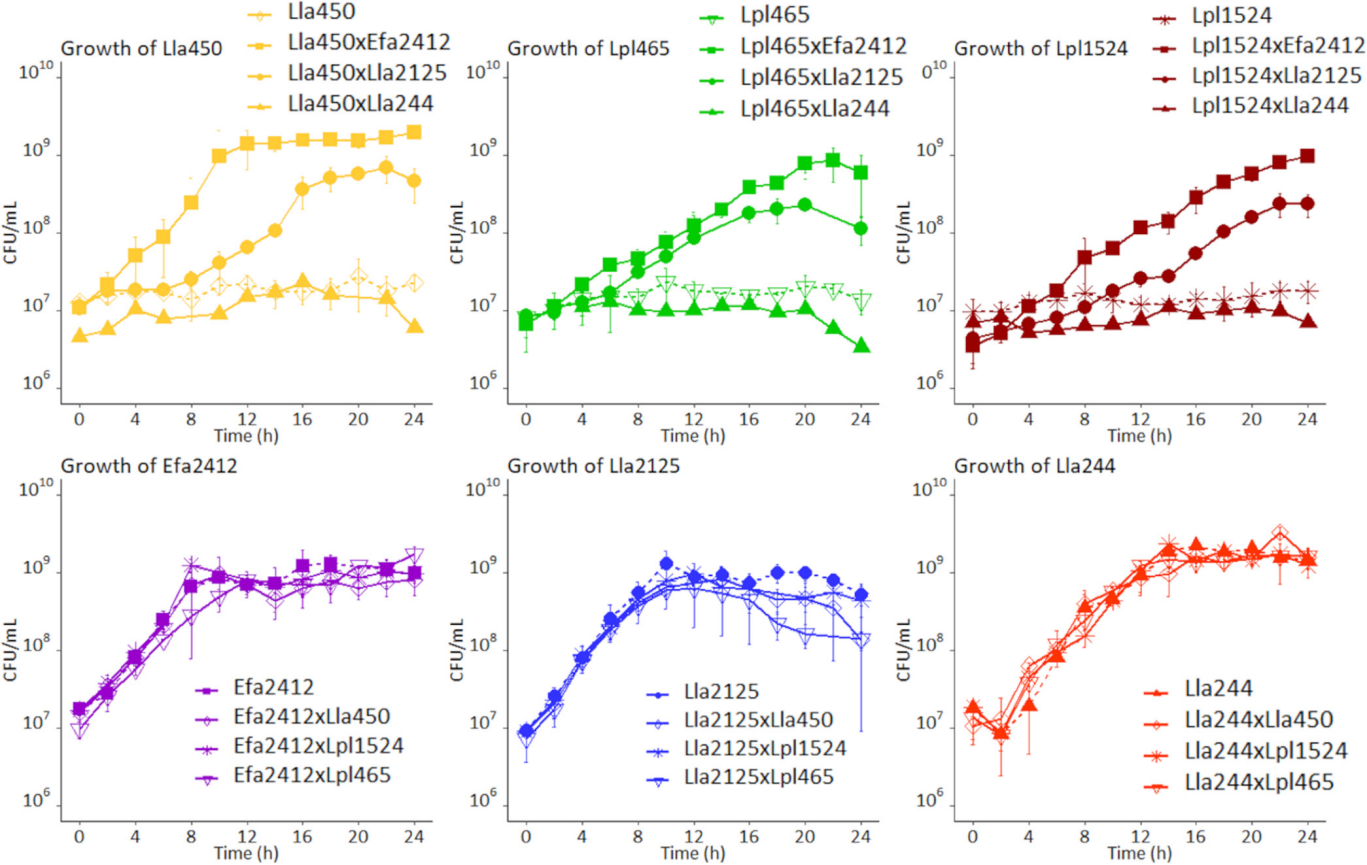
Three different donor stains resulted in three different types of interactions. Growth curves of the six LAB strains used in the study incubated in compartmented chambers at 30°C for 24 h are shown. The proteolytic (donor) strains (bottom) were as follows: Enterococcus faecalis CIRM-BIA2412 (Efa2412), Lactococcus lactis NCDO2125 (Lla2125), and L. lactis CIRM-BIA244 (Lla244), The nonproteolytic (receiver) strains (top) were as follows: L. lactis NCDO2111 (Lla450), *Lactiplantibacillus plantarum* CIRM-BIA465 (Lpl465), and *L. plantarum* CIRM-BIA1524 (Lpl1524), in mono- and cocultures. Each one of the proteolytic strains was associated with one nonproteolytic strain. Bacterial counts were made on MRS agar for *L. plantarum* strains and M17 for the rest of the strains.

Figure 3 shows that the three donor strains, Efa2412, Lla2125, and Lla244, in monocultures (dotted lines, lower portion) grew well in the CDM PROT medium, reaching populations of 9.1 ± 0.2, 9.0 ± 0.1 and 9.3 ± 0.1 log CFU/ml, respectively, after 24 h of culture. In contrast, the three strains L. lactis NCDO2111 (Lla450), *L. plantarum* CIRM-BIA465 (Lpl465), and *L. plantarum* CIRM-BIA1524 (Lpl1524) did not grow in CDM PROT in monocultures (dotted lines, upper portion). They maintained their cultivable populations at the initial level of inoculation after 24 h of culture, and the pH of the medium remained steady.

To validate that the factor limiting the growth of the three nongrowing strains was the nitrogen source, we added 0.5 g/liter of casein tryptone, which supplies casein peptides and free amino acids, to the CDM PROT medium; the new medium is referred to as CDM TRYP. Growth of strains Lla450, Lpl465, and Lpl1524 was then restored after 24 h as their cultivable populations reached 9.1 ± 0.1, 8.8 ± 0.2, and 8.9 ± 0.1 log CFU/ml, respectively. These three strains were then considered nonproteolytic and referred to as receiver strains.

CDM TRYP, in which all receiver strains grew, contained 108.9 mg/liter of FAA and a peptide concentration estimated at 7.7 mg NH_2_/liter (Fig. 2). It was therefore considered a positive control for supporting receiver strain growth. The total nitrogen content of CDM TRYP was lower than that of the three donor strains. Thus, at first glance, all three donor strains would be capable of providing the receiver strains enough nitrogen compounds for their growth.

### Bacterial growth in cocultures.

The maximal counts of the three donor strains, Efa2412, Lla2125, and Lla244, were not impacted by the mode of culture, either mono- or coculture (Fig. 3). They reached in 14 h plateaus of 9.0 ± 0.2 log CFU/ml in 8 h, 9.0 ± 0.2 log CFU/ml in 12 h, and 9.2 ± 0.1 log CFU/ml, respectively. However, the population of Lla2125 decreased significantly after reaching the plateau in coculture with Lla450 and Lpl465.

In coculture with Efa2412, the counts of Lla450, Lpl465, and Lpl1524 reached maxima of 9.0 ± 0.1 log CFU/ml within 10 h, 8.9 ± 0.3 log CFU/ml in 22 h, and 9.0 ± 0.1 log CFU/ml in 24 h of culture, respectively (Fig. 3). In coculture with Lla2125, the counts of Lla450, Lpl465, and Lpl1524 reached maxima of 8.8 ± 0.2 log CFU/ml in 20 h, 8.5 ± 0.6 log CFU/ml in 22 h, and 8.4 ± 0.2 log CFU/ml in 22 h, respectively. The three receivers grew at a higher rate in coculture with the donor strain Efa2412 than with the donor strain Lla2125. In coculture with Lla244, the counts of Lpl1524 remained at the inoculation level of 7.0 ± 0.4 log CFU/ml, whereas the counts of Lla450 and Lpl465 decreased to 6.5 ± 0.1 log CFU/ml (Fig. 3).

To summarize, three types of interactions were observed between the receiver strains and the donor strains, according to the donor strain: strong interactions with Efa2412, weak interactions with Lla2125, and no interaction with Lla244.

We checked that bacterial growth in cocultures performed in compartmented chambers was equivalent to that in direct cocultures. We chose the specific incubation time of 14 h for this verification because it corresponded to the end of exponential growth phase of the donor strains and the middle of the exponential growth phase of the *L. plantarum* receiver strains. This receiver species was chosen to facilitate count in direct culture on both different plate media for donor and receiver. No significant differences were observed between the bacterial counts obtained in direct cocultures and in compartmented chambers. Therefore, we chose to use direct cocultures to study the impact of cocultivation on acidification, carbohydrates hydrolysis, and volatile profiles for the rest of the study.

### Acidification parameters were influenced by the type of the interactions and the intrinsic capacities of the receiver strains.

The acidification rates of the different LAB strains in mono- and cocultures as well as the final pH observed after 24 h of fermentation are shown in Table 1. In monoculture in CDM PROT, the donor strains Lla2125 and Lla244 showed faster acidification than Efa2412. Among the receiver strains, Lla450 showed faster acidification than Lpl465 and Lpl1524.

**TABLE 1:** Maximal acidification rates and pH of CDM PROT after 24 h culture of LAB strains^*a*^

Receiver strain	Maximal acidification rate (dpH/h; absolute value) for indicated donor strain				Final pH after 24 h for indicated donor strain			
	None	Efa2412	Lla2125	Lla244	None	Efa2412	Lla2125	Lla244
None		0.60 CD	1.14 A	1.05 AB		4.20 GH	4.12 F	3.94 AB
Lla450	0.53 D	0.99 B	0.97 B	1.04 AB	4.16 G	4.07 DE	4.10 EF	3.94 AB
Lpl465	0.35 E	0.69 CD	1.11 AB	1.01 AB	4.22 H	3.96 B	4.04 CD	3.93 AB
Lpl1524	0.21 E	0.69 C	1.09 AB	1.02 AB	4.55 I	4.01 C	4.08 DEF	3.91 A

a Donor strains Efa2412, Lla2125, and Lla244 were incubated as monocultures and in cocultures with Lla450, Lpl465, and Lpl1524. Results for the LAB receiver strains Lla450, Lpl465, Lpl1524 in monoculture were obtained in the CDM TRYP, which corresponds to CDM PROT supplemented with 0.5 g/liter of casein tryptone. For strain codes, see Table 4. dpH/h corresponds to the maximal difference in pH obtained in an hour. Different uppercase letters after the numbers indicate significant differences between cultures (*P* < 0.05).

The acidification rates of the cocultures depended on the receiver strains associated with the donor strains. The two *L. plantarum* strains did not impact the acidification rates of the donor strains with which they were cocultured. In contrast, different acidification rates were observed for Lla450 depending on the associated donor strains. Lla450 induced a 65% increase of the maximal acidification rate in coculture with Efa2412, while a 15% decrease was observed with Lla2125 and no change of the acidification rate was observed in coculture with Lla244.

The final pHs in monocultures ranged from 3.94 to 4.55 for Lla244 and Lpl1524, respectively (Table 1). In the three cocultures with the donor strain Efa2412, the final pH was significantly lower than that of the donor strain in monoculture, in particular with the two *L. plantarum* strains. Considering the donor strain Lla2125, only its coculture with Lpl465 significantly decreased the final pH compared to that of the monoculture of the donor strain. Concerning the donor strain Lla244, the final pHs did not differ in mono- and cocultures.

### The type of interactions modulates the carbohydrate consumption.

**FIG 4:**
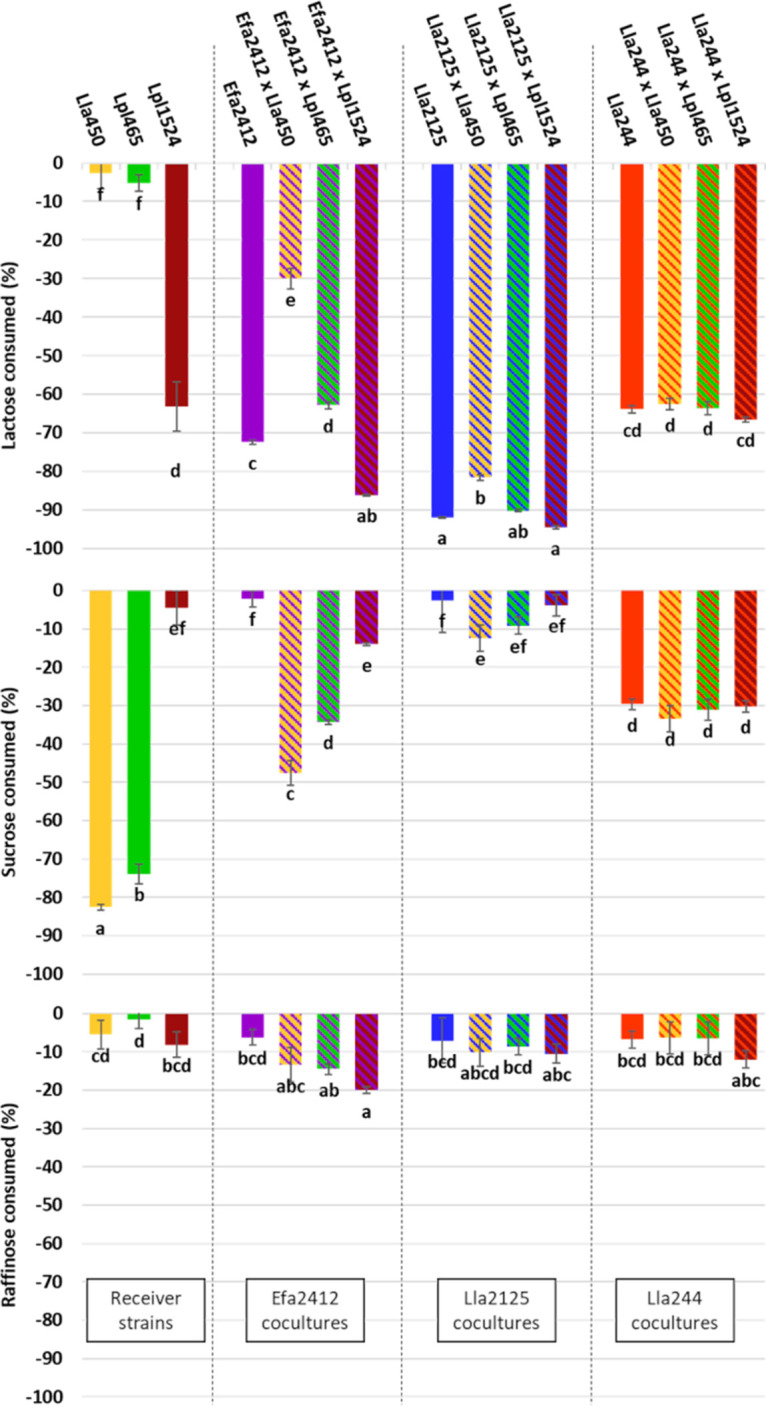
Carbohydrate consumption can vary according to the strains associated. Percentages of lactose, sucrose, and raffinose consumed in mono- and cocultures of LAB donor and receiver strains during 24 h of incubation at 30°C are shown. Percentages were calculated as a ratio of the amount in the carbohydrate after fermentation relative to the amount of the noninoculated media used as a control: CDM PROT was used as the control for the donor strains Efa2412, Lla2125, Lla244 and the cocultures, and CDM TRYP was used for the receiver strains Lla450, Lpl465, and Lpl1524 monocultures. Initial concentrations, in grams per liter, were as follows: lactose, 4.78 ± 0.50; sucrose, 4.03 ± 0.21; and raffinose, 4.63 ± 0.23. Different letters indicate significant differences between cultures (*P* < 0.05).

The consumption of milk carbohydrates, i.e., lactose, and lupin carbohydrates, i.e., raffinose and sucrose, are shown in Fig. 4. Lactose was hydrolyzed by all strains except Lla450, in agreement with the results observed using API gallery (see Materials and Methods). The consumption of lactose in the cocultures with donor strain Efa2412 significantly varied depending on the associated receiver strain. A total of 72% of lactose was consumed with Efa2412 alone, versus 30%, 62%, and 87% in its cocultures with Lla450, Lpl465, and Lpl1524, respectively. Lactose consumption was less impacted by the cocultures involving donor strain Lla2125. There was significantly less lactose consumed when Lla2125 was cocultured with Lla450 (81% of lactose consumed in coculture, versus 90% with Lla2125 in monoculture). No significant differences were observed in the cocultures involving Lla2125 and the two *L. plantarum* strains. The cocultures involving donor strain Lla244 did not change the percentage of lactose consumed (65%).

Sucrose was only consumed by the strains Lla450 (82%), Lpl465 (72%), and Lla244 (30%) in monocultures. Considering the results of the API gallery (see Materials and Methods), a diminution of sucrose was also expected with Efa2412 but was not observed in monoculture. However, its association with Lla450, Lpl465, and Lpl1524 significantly modified sucrose consumption, as 47%, 34%, and 13% were consumed in the respective cocultures, showing the individual carbohydrate consumption by the strains. Likewise, the strain Lla2125 did not consume sucrose from the medium, but the association with Lla450 significantly increased sucrose consumption to 12%. The cocultures involving the donor strain Lla244 did not change the percentage of sucrose consumed.

Concerning raffinose, even if the three receiver strains were capable of hydrolyzing this carbohydrate, as shown by the API gallery results, less than 8% was consumed by these strains in monocultures. The only association that significantly increased raffinose consumption (to 20%) was the coculture of Efa2412 with Lpl1524.

### Volatile compound profiles in direct cocultures.

Among volatile compounds, 12 compounds exhibiting significant changes in concentration in mono- or cocultures compared to the unfermented control samples (fold change >5 or <0.1) were identified (Table 2). They represented five chemical classes—five aldehydes, one sulfur-containing compound, four ketones, one acid, and one alcohol—and they derived from different pathways: five from amino acid catabolism, four from carbohydrate metabolism, and one from free fatty acid catabolism (Table 2). Two linear aliphatic aldehydes, heptanal and hexanal, were 10-fold more concentrated in the control medium than in LAB cultures. Cocultures did not contribute to a further decrease in the concentrations of hexanal and heptanal compared to the monocultures of the donor strains. The 10 other volatile compounds were produced by the LAB strains both in mono- and cocultures. The highest fold changes between cultures and controls varied from ∼3 for benzaldehyde to ∼20,000 for acetoin. The greatest fold changes were observed with donor strain Lla244 and/or the cocultures involving receiver strain Lla450 for most (10 out of 12) of the volatile compounds (Table 2). Although the volatile-compound profiles differed according to the donor and receiver strains, most of the compounds were produced in various amounts by all strains, except 2,3-pentanedione, produced by only two strains (Lla450 and Lla244).

**TABLE 2:** Volatile compounds identified in CDM after 24 h of fermentation at 30°C with the six LAB strains in mono- and cocultures and in unfermented CDM used as controls (CDM TRYP and CDM PROT)^*a*^

Compound (trivial name)	*m/z*	Ab	Identification	Associated odor/flavor^*b*^	CAS no.	LRI	Max fold change^*c*^	Culture associated with the maximal culture/control ratio	Origin
3-Methylbutanal	58	MBT	S, LRI, DB	Fruity	590-86-3	918	905 ± 15	Lla244 × Lla450	cata, Leu
2,3-Butanedione (diacetyl)	42	D	LRI, DB	Buttery	431-03-8	975	39 ± 3	Lla244 × Lla450	metab, C
2,3-Pentanedione	100	PD	LRI, DB	Buttery	600-14-6	1,062	150 ± 23.4	Efa2412 × Lla450	metab, C
Hexanal	44	H	LRI, DB	Grassy	66-25-1	1,080	0.06 ± 0.02	Lla2125	CDM
1-Propanol, 2-methyl	74	MPP	LRI, DB	Fusel	78-83-1	1,114	3,890 ± 240	Lla244 × Lpl465	cata, Val
Heptanal	44	HP	LRI, DB	Green	111-71-7	1,183	0.12 ± 0.06	Lla2125 × Lla450	CDM
2-Hydroxy-3-butanone (acetoin)	88	AC	LRI, DB	Buttery	513-86-0	1,277	22,211 ± 3,014	Lla2125 × Lpl1524	metab, C
2-Nonanone	58	NN	S, LRI, DB	Cheesy	821-55-6	1,383	6.8 ± 0.4	Lla244 × Lla450	cata, FFA
Acetic acid	60	A	S, LRI, DB	Sour	64-19-7	1,476	558 ± 82	Efa2412 × Lla450	metab, C
Benzaldehyde	106	BZH	S, LRI, DB	Nutty	100-52-7	1,535	3.3 ± 0.1	Efa2412 × Lla450	cata, Phe
2-Methylthiolan-3-one	60	MTL	LRI, DB	Sulfurous fruity berry	13679-85-1	1,539	20.6 ± 2.6	Lla244	cata, Met
Benzeneacetaldehyde	91	BZAH	LRI, DB	Honey	122-78-1	1,635	223 ± 7	Lla244	cata, Phe

a Receiver strains Lla450, Lpl465, and Lpl1524 were grown on CDM TRYP, which corresponds to CDM PROT supplemented with 0.5 g/liter of casein tryptone. The selection criterion for the volatile compounds was a culture/control ratio of >5 or < 0.1. Compound name is according to IUPAC (International Union of Pure and Applied Chemistry) nomenclature. CAS, Chemical Abstract Service registry; S, retention time and mass spectrum from standard; LRI, linear retention index; DB, mass spectral data library of the National Institute of Standards and Technology (NIST); Ab, abbreviation, cata, catabolism; metab, metabolism; C, carbohydrate; FFA, free fatty acid. For strain codes, see Table 4.

b Flavor description according to The Good Scents Company Information System.

c Culture/control ratio. Max, maximum.

**FIG 5:**
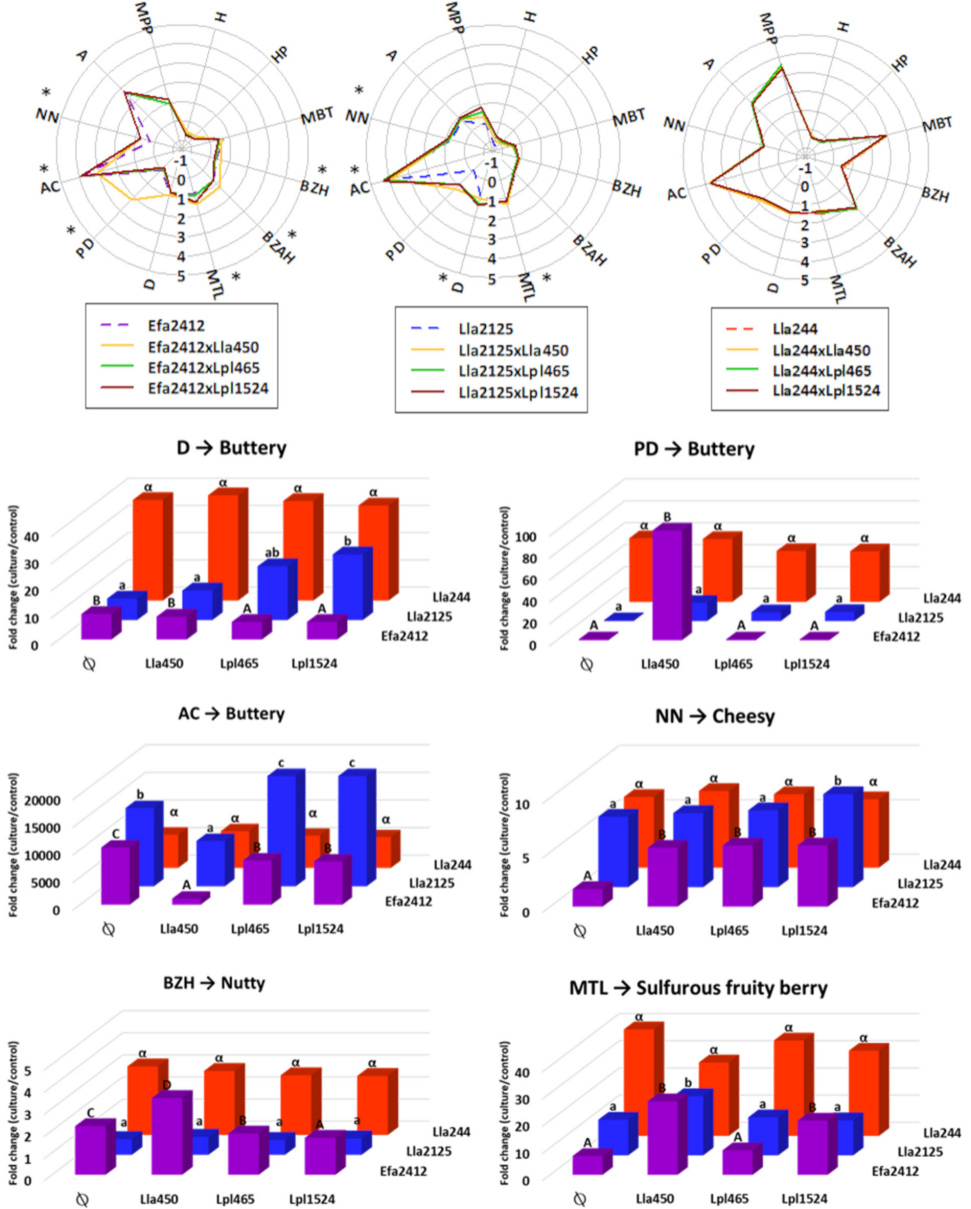
Cocultures increased the concentrations of volatile compounds potentially associated with pleasant flavors. (Top) Radar charts of the abundance ratio (culture to control) of the 12 volatile compounds selected, expressed in log for the LAB donor strains Efa2412 and its cocultures, Lla2125 and its cocultures, and Lla244 and its cocultures, all cultured in CDM PROT. “*” represents the volatile compounds that are significantly increased in at least one coculture compared to the donor strain in monoculture. (Bottom) Fold changes (culture to control) of 6 volatile compounds whose concentrations were significantly increased with cocultures. “Ø” represents the absence of the receiving strain, i.e., the monocultures of the donor strains. For the strain codes, see Table 4. H, hexanal; HP, heptanal; MBT, 3-methylbutanal; BZH, benzaldehyde; BZAH, benzene acetaldehyde; MTL, 2-methylthiolan-3-one; D, diacetyl; PD, 2,3-pentanedione; AC, acetoin; NN, 2-nonanone; A, acetic acid; MPP, 2-methyl-1-propanol.

The global profiles of volatile compounds produced mainly depended on the donor strains, as shown in the radar plots in Fig. 5 (top), which illustrate the fold change in log of the most impacted volatile compounds in mono- and cocultures. The donor strains presented differences in the volatile compounds they produced in monocultures. Globally, Lla2125 produced the lowest level of volatile compounds and Lla244 the highest, especially benzeneacetaldehyde (BZAH), 2-methyl-1-propanol (MPP), and 2,3-pentanedione (PD). Efa2412 produced more benzaldehyde (BZH) and acetic acid (A) than Lla2125.

The association of Efa2412 with each receiver strain led to higher levels of five volatile compounds (PD, 2-nonanone [NN], BZH, BZAH [data not shown], and 2-methylthiolan-3-one [MTL]) than in the monoculture of Efa2412 (Fig. 5, bottom). PD, NN, BZH, BZAH, and MTL were associated with buttery, cheesy, honey, nutty, and sulfurous fruity berry flavors, respectively. Concerning donor strain Lla2125, its coculture with the receiver strains significantly increased the production of four volatile compounds (diacetyl [D], NN, acetoin [AC], and MTL). D and AC were associated with buttery flavor. Concerning Lla244 and its cocultures, there was no significant difference in the volatile compounds produced (Fig. 5).

### Insight on the NH_2_-containing compounds available for the receiver strains.

Each donor strain showed a particular amino acid profile which could influence the growth of the receiver strains. Regarding the FAA released, we focused on the 13 considered essential for the growth of L. lactis (19) and/or *L. plantarum* (20) and compared their contents in the medium after culture of each of the donor strain and in the medium used to grow the receiver strains (CDM TRYP) (Table 3). Four FAA were exhausted in the medium after some cultures. Hence, there was no Met or Arg left after the culture of the three donor strains, while both of these FAA were present in CDM TRYP. In addition, Efa2412 did not leave Tyr, while the two other donor strains did not leave Trp and Lla244 did not leave Ile. The concentrations of the FAA present also varied according to the donor strain: Efa2412 monoculture contained significantly more Val, His, Ile, Leu, Phe, and Trp than the two other donor strain monocultures and CDM TRYP; Lla2125 monoculture was characterized by higher concentrations of Asp, Thr, Ser, Glu, and Tyr.

**TABLE 3:** Concentrations of the 13 essential amino acids after 24 h of fermentation of the CDM PROT by the donor strains Efa2412, Lla2125, and Lla244 and present in the CDM TRYP prior to the culture with the receiver strains^*a*^

Amino acid	Concn (%) of amino acid after fermentation by:			Concn in CDM TRYP
	Efa2412	Lla2125	Lla244	
Asparagine	6.9 (1.7)	**11.0** (2.7)	4.7 (1.3)	1.9
Threonine	4.8 (2.7)	**7.3** (4.2)	2.1 (1.2)	2.4
Serine	2.3 (0.9)	**6.6** (2.7)	1.4 (0.6)	2.4
Glutamic acid	64.7 (6.2)	**111.9** (10.7)	61.9 (5.9)	1.0
Valine	**13.1** (5.8)	5.9 (2.6)	1.7 (0.7)	5.1
Methionine	0	0	0	**3.9**
Histidine	**7.4** (7.2)	5.4 (5.2)	5.3 (5.1)	1.5
Isoleucine	**5.7** (2.8)	3.2 (1.6)	0	3.4
Leucine	**33.5** (9.0)	10.0 (2.7)	2.7 (0.7)	22.0
Tyrosine	0	**13.9** (5.5)	10.8 (4.3)	2.7
Phenylalanine	**24.5** (11.7)	8.8 (4.2)	8.7 (4.2)	12.0
Arginine	0	0	0	**15.3**
Tryptophan	**10.1** (ND)	0	0	0

a Concentrations are in milligrams per liter. Percentages, in parentheses, represent the quantity of each amino acid released from milk and/or lupin proteins of the CDM. For strain codes, see Table 4. The significantly highest concentrations for each amino acid are shown in bold. ND, not determined.

In four out of the six cocultures with donor strains Efa2412 and Lla2125, the overall amount of FAA was significantly smaller than the one in the donor strain monocultures: 65%, 9% 11%, and 8% with Efa2412 × Lla450, Efa2412 × Lpl465, Lla2125 × Lla450, and Lla2125 × Lpl465, respectively. Arg, Trp, and Ile were no longer detectable after the culture of Lla450 with Efa2412 and could thus have limited the growth of both donor and receiver strains. The remaining essential FAA were detectable (>1 mg/liter) in all nine cocultures after fermentation, suggesting that they were not limiting (data not shown).

The concentrations in peptides were significantly reduced in the cocultures of donor strain Efa2412 with each receiver (54%, 16.8%, and 19.3% with Lla450, Lpl465, and Lpl1524, respectively) (data not shown). The ratio of FAA to total NH_2_ compounds tended to decrease in the cultures involving Lla450 and, in contrast, to increase in the cultures involving Lpl465 and Lpl1524, indicating that the receiver L. lactis strain tested preferentially consumed amino acids, whereas the *L. plantarum* strains preferentially consumed peptides.

### The absence of interaction is not due to growth inhibitor production or isoleucine deficiency.

**FIG 6:**
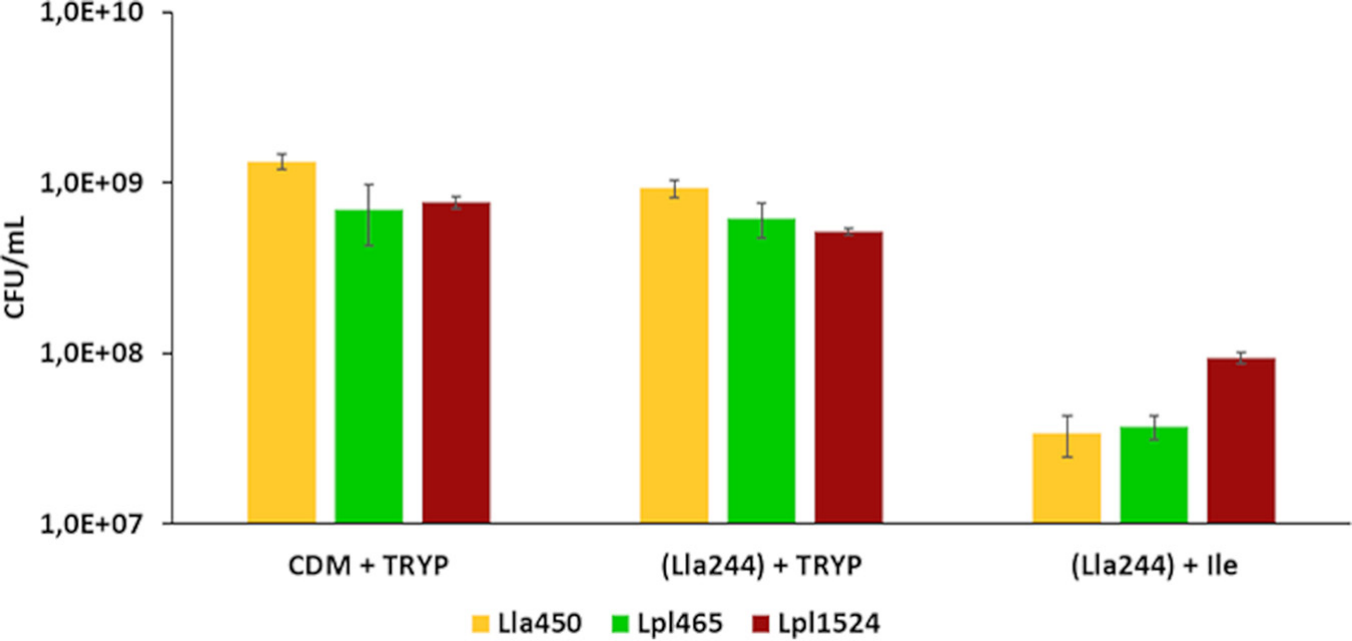
The absence of interaction is not related to a growth inhibitor factor or a deficiency in isoleucine. Shown are bacterial counts of the three receiver strains cultured in CDM PROT supplemented with 0.5 g/liter of casein tryptone (CDM + TRYP), supernatant of donor strain Lla244 cultured in CDM PROT for 24 h and supplemented with 0.5 g/liter of casein tryptone [(Lla244) + TRYP], and supernatant of donor strain Lla244 cultured in CDM PROT for 24 h and supplemented with 5 mg/liter of isoleucine [(Lla244) + Ile].

Regarding the lack of interaction between donor strain Lla244 and the receiver strains, two hypotheses were made: either the production of a growth inhibitor or a deficiency in available Ile was the cause. Figure 6 shows that all receiver strains reached 10^9^ CFU/ml within 24 h of culture in CDM TRYP. The receiver strains grew in the culture supernatant of Lla244 supplemented with casein tryptone: bacterial counts exceeded 5.10^8^ CFU/ml and were similar to the bacterial counts observed in CDM TRYP. This result suggests that no growth inhibitor was produced by the donor strain Lla244. The receiver strains grew but did not reach 10^8^ CFU/ml when cultured in the culture supernatant of Lla244 supplemented with 5 mg/liter of Ile. This result suggests that isoleucine was not the only limiting growth factor explaining why no interactions were observed between the donor strain Lla244 and the receiver strains.

### Expression of the functional outputs by the different cocultures summarized by principal-component analyses (PCA) on the whole data set.

**FIG 7:**
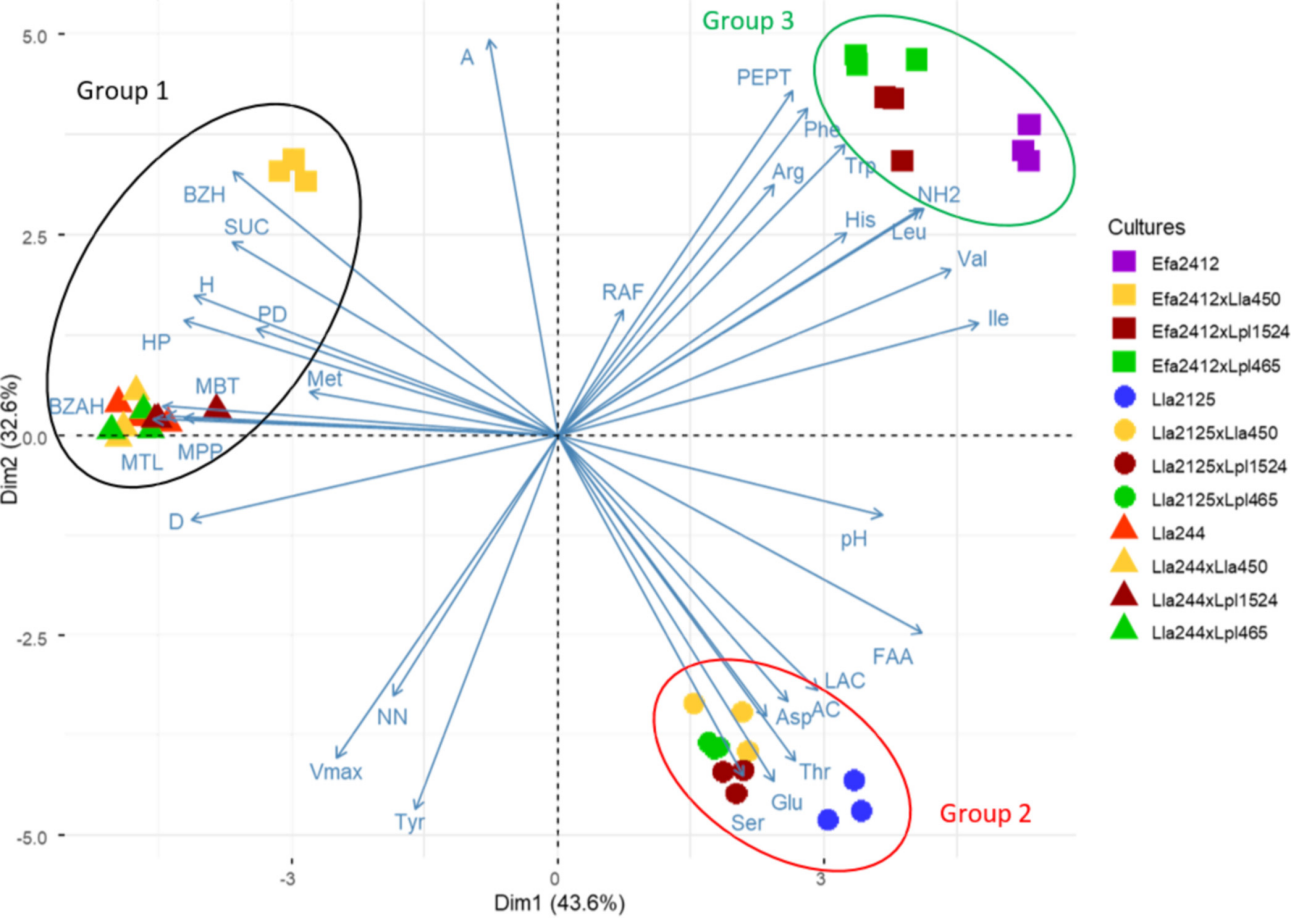
Parameters differentiating the cultures. Shown is a biplot of the first two dimensions of principal-component analysis performed using 33 variables: 12 selected volatile compounds expressed as a culture/control (noninoculated CDM PROT) ratio; 2 acidification parameters, maximal acidification rate (*V*_max_) and final pH; 3 carbohydrates, expressed as the percentage of carbohydrate consumed (lactose [LAC], sucrose [SUC], and raffinose [RAF]), and 16 variables that describe the content in free amino acids and peptides: 13 essential amino acids, free NH_2_ groups, concentration in free amino acids (FAA), and concentration in peptides (PEPT) measured in CDM PROT fermented by the monocultures of the strains Efa2412, Lla2125, and Lla244 and their cocultures with three receiver strains Lla450, Lpl465, and Lpl1524. Replicate experiments are represented using the same symbols. For volatile-compound abbreviations, see Table 2. The ellipses result from a hierarchical clustering performed on the PCA data set. For strain codes, see Table 4.

A total of 33 variables of four categories were selected to characterize the functional outputs of the three monocultures of donor strains and the nine cocultures: two acidification parameters, consumption of three carbohydrates, volatile compounds (*n* = 12), and the nitrogen composition of the medium after 24 h (including the content in each of the 13 amino acids known to be essential to LAB, global content in FAA, peptides, and the percentage of FAA). The first and second dimensions accounted for 43.6% and 32.6% of total variance, respectively (Fig. 7). The three replicates of cultures appeared all colocalized, which underlines the good reproducibility of the experiments. In most cases, the donor strain was grouped with its three cocultures, except in one case (Efa2412 associated with Lla450).

Three groups were distinguished by hierarchical clustering. Group 1 gathered Lla244 and its cocultures and the coculture Efa2412 with Lla450 and was characterized (*P* < 10^−5^) by high concentrations of most of the volatile compounds (BZH, heptanal [HP], MTL, BZAH, 3-methylbutanal [MBT], hexanal [H], 2-methyl-1-propanol [MPP], and D), low FAA concentrations (more specifically, Ile and Val), and a higher sucrose consumption. Group 2 gathered Lla2125 and its cocultures and was characterized (*P* < 10^−5^) by high concentrations in some amino acids (Glu, Thr, Asn, and Ser) and AC and low concentrations of some volatile compounds (A and BZH). Group 3 gathered Efa2412 and its two cocultures with *L. plantarum* strains and was characterized (*P* < 10^−5^) by high concentrations of branched-chain (Leu, Val, and Ile) and aromatic (Phe and Trp) amino acids and His and globally of free NH_2_-containing compounds (more specifically peptides), low acidification rates, and low Tyr concentrations.

Globally, cocultures of donor strains Lla2125 and Efa2412 showed lower scores on the first dimension than the corresponding monoculture of the donor strain, indicating that they contained less sucrose and more lactose, had a lower pH, and contained fewer NH_2_-containing compounds (FAA and peptides).

## DISCUSSION

This study aimed to promote positive interactions based on nitrogen nutritional dependencies between LAB strains in coculture. This was done in a context of designing starters of new (mixed) food fermentation and seeking consequences of such positive interactions for the functional outputs of the fermented media.

McCully et al. (21) previously determined that a nitrogen starvation response is important for a stable coexistence between Escherichia coli and Rhodopseudomonas palustris. Thus, we first conceived a chemically defined medium, called CDM PROT, which contained proteins as the sole nitrogen source, so as to control the nutritional interactions between strains and allow only proteolytic strains to grow. A similar strategy was used by Ponomarova et al. (11) to study the interactions between yeast and LAB strains. Selecting a medium supporting the growth of microorganisms in cocultures but not in monocultures allowed evidencing nutrient cross-feeding. While S. cerevisiae growth varied very little between monoculture and coculture with LAB in the CDM used, both L. lactis and *L. plantarum* could grow only when cocultured with the yeast, suggesting metabolic dependencies. In our study, we used a mix of milk and lupin proteins as the sole nitrogen source and a mix of lactose, sucrose, and raffinose as the carbon source, to mimic the content of a mix of milk and lupin. This medium thus both facilitated the study of LAB interactions and provided some insight on the outputs that could be obtained by fermenting a more complex and mixed food matrix. As expected, only the three proteolytic strains grew in CDM PROT, whereas none of the three nonproteolytic strains tested did. The growth of the latter was restored by supplementing the medium with a casein hydrolysate, which demonstrates that the lack of available nitrogen nutrients was the only factor that prevented their growth in CDM PROT. In cocultures of pairwise proteolytic and nonproteolytic strains, the former, qualified as donors, were then expected to provide the latter, qualified as receivers, with nitrogen nutrients.

We chose two complementary approaches to explore the interactions between donor and receiver strains. First, cocultures in compartmented chambers facilitated the enumeration of each strain without needing to develop selective enumeration media, in particular in the case of the L. lactis*/*L. lactis cocultures. These chambers allowed us to quickly establish whether both strains grew, when they started to grow, and whether the interactions required or not a physical contact between the strains. In their review on coculture systems, Goers et al. (22) suggested that such devices were excellent tools to explore cell-cell interactions. The kinetics of diffusion of metabolites from one compartment to the other, e.g., nitrogen nutrients and lactic acid, which are expected to enhance or inhibit the growth of the receiver strains (23), did not seem to alter the donor/receiver interactions, since bacterial counts were identical after 14 h in compartmented chambers and in direct cocultures. Second, direct cocultures were chosen to investigate the functional outputs that resulted from LAB metabolism, because they are closer to food fermentation than cocultures in compartmented chambers. Three main outputs were targeted: the acidification rate, which should be high enough for economic and sanitary reasons; the consumption of lactose and raffinose, because these carbohydrates can induce intestinal discomfort; and the production of aroma compounds susceptible to desirably influence food flavor.

Three cases of interactions were observed, which depended only on the donor strains and not on receiver strains tested. First, cocultured with the donor strain E. faecalis CIRM-BIA2412 (Efa2412), the receiver strains quickly started to grow and reached high counts (above 10^9^ CFU/ml), showing strong interactions. Second, cocultured with the donor strain L. lactis NCDO2125 (Lla2125), the receivers grew but stayed below 10^9^ CFU/ml, suggesting weaker interactions. Third, cocultured with the donor strain L. lactis CIRM-BIA244 (Lla244), none of the receivers grew, suggesting the absence of positive interactions. Concerning donor strains, their maximal growth in monoculture was similar to that in coculture (Fig. 3), implying that the positive interactions observed were commensalistic (8), i.e., that the fitness of receivers increased with no apparent cost or benefit for the donors. These differences of interactions could be explained by different factors. The proteolytic activity of the donors probably had likely the main impact in terms of quantity and nature of peptides and free amino acids released (Fig. 2). This also could be balanced by the respective nitrogen nutritional requirements of donors and receivers, as well as their kinetics of growth that could also influence the interactions observed (Fig. 3), as developed below.

Regarding the proteolytic activity, donor strain Efa2412 could be qualified as a “model” donor strain compared to the two other donors, providing the receivers with high concentrations both of several free amino acids (Trp, Leu, Val, Phe, Ile, and Arg) and of peptides (Fig. 2 and 7). Among the three donors, Lla244 liberated the smallest amounts of nitrogen nutrients, but the concentration of FAA exceeded that of the control medium that contained 0.5 g/liter casein hydrolysate and the concentration of peptides exceeded that produced by Lla2125. It was thus unexpected that receiver strains, which grew in the control medium and in the coculture with Lla2125, did not grow in coculture with Lla244. We first hypothesized that Lla244 would produce a growth inhibitor such as a bacteriocin, but this was invalidated by the results observed in sequential cultures, which showed that receiver strains grew in the culture supernatant of Lla244 supplemented with 0.5 g/liter of casein hydrolysate (Fig. 6). We also hypothesized that Ile concentration limited receiver growth, since Ile was the only essential amino acid lacking in Lla244 monoculture (Table 3). This hypothesis was also ruled out since Ile addition did not restore the growth of the two *L. plantarum* strains in direct coculture with Lla244 (Fig. 6). These results suggest that the nutritional dependencies based on nitrogen sources rely on both the nature and concentration of nitrogen nutrients.

The nutritional requirements and preferences for peptides or FAA of both donor and receiver strains can also modulate their interactions. In our study, the comparison between FAA and peptide uptakes by the receiver strains suggests that the L. lactis receiver had no significant preference, whereas the two *L. plantarum* strains preferred peptides to FAA (data not shown). Such a preference for peptides by an *L. plantarum* strain was demonstrated by Saguir et al. (24), who observed that dipeptides were more effective than FAA in sustaining its growth under nutritional stress conditions. This is also in agreement with a higher number of amino acids required for the growth of the species *L. plantarum* than for L. lactis and E. faecalis (20, 25, 26).

Finally, the growth kinetics of donor and receiver strains could also have influenced the interactions observed in the present study. The two *L. plantarum* receivers grew slower than the three donors (Fig. 3) and thus could have been inhibited in coculture by the lactic acid early produced by the donor strain. However, they can support lower pH (27) and thus keep growing even after the donor strain stopped growing. The amensal interactions observed between Lla244 and the receiving strains may also be due to the faster acidification and lower final pH obtained with this donor strain than with the two others (Table 1). To counteract such inhibitions, the inoculation ratios of the donors/receivers associated could be adjusted by increasing the initial counts of the receiver, and/or the donor strains could be chosen among strains that do not exhibit too high an acidification rate or reduce pH to values too low, as is the case for donor Efa2412 compared to donor Lla2125.

The stronger the interactions, the more the outputs observed in cocultures differed from the ones observed in monoculture of the donor strains. In cocultures with donor strain Efa2412, which induced the strongest interactions, all the functional outputs characterized in this study were impacted: the rate and degree of acidification, the consumption of three carbohydrates, and the production of some aroma compounds. Compared to the monoculture Efa2412, a lower final pH was observed in the cocultures with the two *L. plantarum* receivers, a higher acidification rate with Lla450 receiver, and lower final concentrations in lactose, sucrose, and/or raffinose, depending on the receivers. The maximum consumption of raffinose was only 20% in CDM, although the three receiver strains were capable of using it, as shown with the API 50CHL gallery results (Table 4). This apparent discrepancy most likely results from preferences between the three carbohydrates contained in CDM, i.e., lactose, sucrose, and raffinose. In agreement with our results, lactose and/or sucrose was preferentially used, compared to raffinose, by nearly 300 LAB strains of 25 different species grown in soy juice (28). Concerning sucrose and lactose preference, Efa2412 and Lpl1524 preferred sucrose over lactose, whereas Lpl465 preferred lactose over sucrose. The volatile compound profile was also markedly impacted in the cocultures with donor Efa2412, with 5 out of the 12 aroma compounds significantly more produced associated with varied potentially desirable aroma: acetoin (milky), 2-nonanone (fruity-cheesy), 2,3-pentanedione (sweet-caramel-buttery), benzaldehyde (almond), and 2-methylthiolan-3-one (fruity). In cocultures with the second donor strain, Lla2125, which induced only weak interactions, smaller impacts on the functional outputs were observed. Compared to the monoculture of Lla2125, a lower final pH was observed in the cocultures of the receiver Lpl465, and 4 out of the 12 aroma compounds were produced in higher concentrations: 2-nonanone (fruity-cheesy), 2,3-pentanedione (sweet-caramel-buttery), 2-methylthiolan-3-one (fruity), and diacetyl (buttery). Finally, in cocultures with the donor strain Lla244, in which there was no interaction, no further functional outputs were observed compared to the monoculture of this strain.

**TABLE 4:** Origin and characteristics of the strains used for proteolytic activity and carbohydrate consumption using API 50CHL galleries^*a*^

Genus	Species	Strain no.	Origin	Strain code	PI (%)	LAC	SUC	RAF
*Enterococcus*	*faecalis*	CIRM-BIA2412	NA	Efa2412	10.8	+	+	−
*Lactococcus*	*lactis*	NCDO2125	Termite gut	Lla2125	5.5	+	−	−
*Lactococcus*	*lactis*	CIRM-BIA244	Raw milk	Lla244	3.6	+	+	−
*Lactococcus*	*lactis*	NCDO2111	Pea	Lla450	0	−	+	+
*Lactiplantibacillus*	*plantarum*	CIRM-BIA465	Sauerkraut	Lpl465	0	+	+	+
*Lactiplantibacillus*	*plantarum*	CIRM-BIA1524	Silage	Lpl1524	0	+	±	+

a LAC, lactose; SUC, sucrose; RAF, raffinose; PI, proteolytic indices, determined after 24 h of culture in a chemically defined medium containing a mix of caseins and lupin proteins, expressed as a percentage of free NH_2_-containing compounds liberated relative to the calculated maximal amino groups that can be released. NA, nonavailable data.

Many LAB properties are strain dependent (29), and consequently, so are the changes in the functional outputs observed. This gives great opportunity to choose receivers able to modulate the functional outputs targeted. In our study, for example, the growth of receiver Lla450 increased the concentration of some desirable aroma compounds, such as 2,3-pentanedione and 2-methylthiolan-3-one, in coculture. The two *L. plantarum* receivers led to a lower final pH, while receiver Lpl1524 was associated with a decrease in raffinose content. Although residual raffinose remained in the medium, the raffinose hydrolysis activity of Lpl1524 (α-galactosidase) may also remain active in the intestine, as observed for lactase activity (β-galactosidase) after yogurt ingestion (30). In conclusion, our attempt to enforce nitrogen-based nutritional dependencies between LAB strains did not necessarily ensure positive interactions. The resulting functional outputs of fermented media depend on the strength of the interactions binding the LAB strains in coculture. We showed that the amount and the nature of the FAA and peptides released by donor strains impacted the growth of receivers. Further investigation of the peptides produced and consumed would be required to better understand the interactions observed in this study. Omic studies such as transcriptomics, proteomics, peptidomics, and/or metabolomics studies could also be of great interest to investigate the mechanisms of the interactions observed (31, 32). Genetic insight could also be useful to manipulate the genomes to confirm the mechanisms observed. To better understand complex microbial communities, future studies are required, including an increased genetic diversity by adding multiple strains of different genera and species.

## MATERIALS AND METHODS

### Bacterial collection.

Six mesophilic LAB strains were tested in the following experiments: two *Lactiplantibacillus plantarum*, one Lactococcus lactis, and one Enterococcus faecalis strains belonging to the collection of the International Centre for Microbial Resources (CIRM-BIA), dedicated to bacteria of food interest, (INRAE Rennes, France [https://www6.rennes.inrae.fr/stlo_eng/]), and two L. lactis strains from the National Collection of Dairy Organisms (NCDO; now NC of Food Bacteria; Berkshire, UK). The strains were selected on their proteolytic capacities in order to either donor strains able to furnish peptides from the medium and receiver strains unable to grow without an external source of peptides or free amino acids.

### Composition of the chemically defined medium.

The chemically defined medium (CDM) was developed in order to fulfill all the nutritional requirements of lactic acid bacteria (mainly lactococci and lactiplantibacilli) (33) in terms of vitamins, mineral salts, and nucleic acids. The nitrogen source was solely in the form of protein, thus limiting access to amino acids to proteolytic strains. The protein and carbon resources were chosen to mimic the composition of both resources: lactose and caseins for the milk part and sucrose, raffinose, and lupin proteins for the legume part.

The final composition of the medium used was as follows. The buffer was K_2_HPO_4_/KH_2_PO_4_ (50 mM, pH 6.9 ± 0.1). Sugars included lactose, sucrose, and raffinose (all at 5 g/liter and all from Sigma-Aldrich, Munich, Germany). The nitrogen source was sodium caseinate (2.5 g/liter; Eurial, Nantes, France) and lupin protein isolate (LPI; 2.5 g/liter; homemade), obtained from Protilup 450 flour (Lup’ingrédient, Martigne-Ferchaud, France) by precipitating the proteins at pH 4.6 (15). Mineral salts included CaCl_2_ (25 mg/liter; Merck, Darmstadt, Germany), Cl_2_Co (1 mg/liter; Sigma-Aldrich), CuCl_2_·2H_2_O (4 mg/liter; Merck), MgCl_2_·6H_2_O (25 mg/liter) (Carlo Erba, Val-de-Reuil, France), (NH_4_)_6_Mo_7_O_24_·4H_2_O (1 mg/liter; Merck), ZnSO_4_·7H_2_O (1 mg/liter; Sigma-Aldrich), MnSO_4_ (100 mg/liter; Merck), and FeSO_4_ (5 mg/liter; Merck). Vitamins included riboflavin (3 mg/liter; Sigma-Aldrich), nicotinic acid (3 mg/liter; Sigma-Aldrich), calcium pantothenate (3 mg/liter; Sigma-Aldrich), pyridoxine (1 mg/liter; Sigma-Aldrich), biotin (0.5 mg/liter; Sigma-Aldrich), folic acid (1 mg/liter; Sigma-Aldrich), thiamine HCl (0.5 mg/liter; Sigma-Aldrich), *p*-aminobenzoic acid (1 mg/liter; Sigma-Aldrich), and pyridoxal HCl (1 mg/liter; Sigma-Aldrich). Nucleic acids included adenosine (10 mg/liter; Sigma-Aldrich), guanine (10 mg/liter; Sigma-Aldrich), uracil (10 mg/liter; Sigma-Aldrich), inosine (10 mg/liter; Sigma-Aldrich), orotic acid (10 mg/liter; Sigma-Aldrich), and thymidine (10 mg/liter; Sigma-Aldrich). Fat included Tween 80 (0.5 g/liter; Sigma-Aldrich).

The protein fraction was prepared separately from the other constituents. A 2-fold-concentrated solution of lupin proteins and caseins was prepared in osmosed water and sterilized by autoclaving at 115°C for 20 min. In parallel, a 2-fold-concentrated solution of vitamins, mineral salts, sugars, Tween 80, and buffer was sterilized by filtration through a 0.2-μm polyethersulfone (PES) membrane filter (Thermo Scientific, Waltham, MA). Then these two solutions were mixed to constitute CDM PROT, which was stored at 4°C for less than 1 month and isolated from the light with aluminum foil.

A supplementary CDM, referred to as CDM TRYP, was also prepared to enable the growth of the nonproteolytic strains. It consists of the same CDM preparation, supplemented with 0.5 g/liter of casein tryptone (Biokar, Allonne, France). The receiver strains were always grown in CDM TRYP when tested in monocultures.

Two media were used to verify the absence of growth inhibitor in Lactococcus lactis CIRM-BIA244 cultures and to verify the deficiency of isoleucine in these cultures. The two media first consisted of the culture supernatant of L. lactis CIRM-BIA244 in CDM PROT incubated for 24 h at 30°C, harvested by centrifugation at 5,000 × *g* for 10 min, and adjusted to pH 7 with NaOH (5 N). The supernatant was sterilized using a 0.22-μm filter. For the medium used for the verification of the absence of growth inhibitor, a sterile 10% tryptone solution was added to reach a final concentration of 0.5 g/liter. For the medium used for the verification of the deficiency of isoleucine, a sterile solution of isoleucine (10%) was added to reach a final concentration of 5 mg/liter.

### Culture conditions.

**(i) Precultures.** LAB strains were conserved in cryotubes at −80°C. One cryotube was used for each replicate culture. Bacteria were cultured once in a rich medium: M17 for lactococci (34) and de Man Rogosa and Sharpe broth (MRS) for lactobacilli (35). Two precultures were made in the CDM PROT in order to adapt the proteolytic strains to the medium. The receiver strains that are not proteolytic were cultured on CDM TRYP to enable their growth.

### (ii) Compartmented chamber setup.

The compartmented chambers (C.P.I.L., Issoire, France), similar to the one used by Paul et al. (36), had a usable volume of 25 ml (30 ml total) and were fixed together with a clamp. O-rings were placed between the two chambers so as to guarantee the sealing of the system. The membrane used to separate the two compartments was a 0.4-μm polycarbonate membrane (Isopore; Merck Millipore, Darmstadt, Germany). Prior to inoculation, the chambers as well as the filters were sterilized at 121°C for 15 min. O-rings were decontaminated using ethanol and rinsed with sterile deionized water. The whole setup was installed under sterile conditions.

### (iii) Cocultures in compartmented chambers and direct cocultures.

The three proteolytic strains, Lla244, Lla2125, and Efa2412, referred to as donors, were associated in direct cocultures or in compartmented chambers, each with each of three nonproteolytic strains, Lla450, Lpl465, and Lpl1524, referred to as receivers, thus generating nine pairs of donor/receiver strains. In all cases, the strains were then inoculated at a total count of 10^7^ CFU/ml of CDM PROT or CDM TRYP, either in chambers (25 ml in each chamber) or in Falcon tubes of 15 ml. One Falcon tube per time and several chambers were used so that volumes would not be limiting for the sampling (up to 6 samples of 2 ml were taken per chamber).

In the chambers, the strains were incubated at 30°C for 24 h, with low orbital shaking (65 rpm) in order to limit the medium aeration while improving diffusion. The CDM were also incubated as an unfermented control.

Culturable bacterial counts were determined with appropriate diluted suspensions of the samples in 1 g/liter of tryptone plus 8.5 g/liter of NaCl solution in microplates (37). Lactococci and enterococci were incubated for 24 to 48 h under aerobic conditions in M17-glucose, and lactiplantibacilli were cultured for 48 h anaerobically using CO_2_ generators (BD Biosciences, San Jose, CA) in MRS, both at 30°C.

Direct cocultures were incubated at 30°C for 24 h, and samples were then collected for further carbohydrate and volatile-compound analyses. The growth of donor strains was controlled after they reached their maximum populations, i.e., after ∼14 h of culture for both mono- and cocultures, using a selective medium constituted by the CDM PROT with 12 g/liter of agar, incubated for 72 h. Cultures were made in triplicates, independently.

### Biochemical analyses.

**(i) Acidification parameters.** Acidification kinetics were established in direct cocultures, using a wireless iCinac (AMS, Frépillon, France) to estimate the maximal acidification rates by calculating the slope between pH 5.5 and pH 5 in all graphs. The final pH was measured using a pH meter (WTW, Weilheim, Germany) after 24 h of incubation.

### (ii) Carbohydrate analysis.

A total of 100 μl of sulfosalicylic acid (2.3 M) was added to 1 ml of sample for deproteinization. The samples were then placed at 4°C for 1 h prior to centrifugation at 10,000 × *g* for 15 min. The supernatants were then filtered through a 0.22-μm membrane and stored at −20°C prior to analyses. Lactose, sucrose, and raffinose were quantified by anion-exchange chromatography using an ICS-5000+ Dionex system (Thermo Electron SA, Courtaboeuf France) fitted with a CarboPac PA210-4μm (2 by 150 mm) analytical column (preceded by a corresponding guard column [2 by 30 mm]). The eluent used was KOH generated with the eluent source Dionex EGC 500 KOH+ eluent generator cartridge and ultrapure water from the Arium Pro system (Sartorius). Cation exchange–high-performance liquid chromatography (CE-HPLC) was run at 30°C with a flow rate of 0.2 ml/min and the gradient was as follows: initial conditions, 13 mM KOH maintained for 32 min, and then a linear rise to 42 min up to 100 mM KOH maintained from 42 to 52 min, followed by reversion to the initial conditions with a linear decrease from 52 to 60 min. Quantification was performed with an external calibrating using carbohydrate standards (Sigma-Aldrich) prepared at 2, 5, 10, 20, and 40 mg/liter (linearity range).

### (iii) Volatile-compound analysis.

Volatile compounds were extracted using a Turbomatrix HS-40 trap automatic headspace sampler and analyzed using a Clarus 680 gas chromatograph coupled to a Clarus 600T quadrupole mass spectrometer, operated within a mass range of *m/z* 29 to *m/z* 206 and ionization impact of 70 eV (Perkin Elmer, Courtaboeuf, France) as detailed previously (38). In brief, 2.5-g quantities of samples were placed in 20-ml Perkin Elmer vials and stored at −80°C prior to analysis. Compounds were eluted on an Elite WAX ETR column (30 m by 0.25 mm by 0.25 μm; Perkin Elmer, Waltham, MA), with helium as the mobile phase, under the following conditions: initial temperature 35°C maintained for 10 min and then increased at 5°C/min up to 230°C. Volatile compounds were identified using the National Institute of Standards and Technology (NIST) 2008 mass spectral library data (Scientific Instrument Services, Ringoes, NJ) and by comparing the retention indexes and mass spectral data of standards. Volatiles were semiquantified from the abundance of one specific mass fragment (*m/z*), in arbitrary units. Mass spectrometry (MS) data were processed using XCMS on R software (39). The full width at half maximum was set to 5, the maximum number of peaks per ion to 100, the interval of *m/z* value for peak picking to 0.4, the signal-to-noise ratio threshold to 6, the group bandwidth to 3, and the minimum to 0.4. The other parameters were those by default. The results are expressed as fold change, i.e., ratio between the concentration in cultures and in unfermented CDM used as controls.

### (iv) Amino acid analysis.

Free amino acid content was determined after deproteinization as described for the carbohydrate analyses above. After filtration through a 0.45-μm pore size membrane (Sartorius, Palaiseau, France), the supernatants were diluted three times with 0.2 M lithium citrate buffer (pH 2.2) prior to injection. Amino acids were analyzed using cation exchange chromatography on a Biochrom 30 AA analyzer (Biochrom Ltd., Cambridge, UK) according to the method of Spackman et al. (40) with lithium citrate buffers as eluents and ninhydrin as a post-column reaction system.

Total amino acid content in the LPI and caseinate was determined with a complete hydrolysis of the proteins with concentrated HCl. These samples were hydrolyzed at 110°C for 24 h in the presence of 6 N HCl using 1.5 ml of acid for an equivalent of 2 mg of total nitrogen. Tubes were then dried and samples were resuspended in 5 ml of 0.2 M lithium citrate buffer (pH 2.2) prior to injection on the Biochrom 30 AA analyzer. For sulfurous amino acids, samples were initially oxidized overnight at 0°C with 2 ml of performic acid then dried before the acid hydrolysis.

### (v) Free amino group dosage and proteolytic index calculation.

The changes in the amount of nitrogen compounds, i.e., peptides and free amino acids, present in the CDM fermented or not after 24 h of incubation were measured in triplicates using the *o*-phtaldialdehyde (OPA) method of Church et al. (41) adapted to microplates. The proteins were precipitated prior to the assay by half-diluting samples with 2% (wt/wt) trichloroacetic acid (final concentration) for allowing the free NH_2_ groups present at the N-terminal extremity of the peptides and amino acids to react with the OPA and the β-mercaptoethanol and to be preferentially detected by spectrophotometry at 340 nm. The results were expressed as milligrams of free NH_2_ per milliliter. Methionine was used as a standard.

The proteolytic index (PI) represents the number of free amino groups relative to the total amino groups. It is therefore available for calculating the ratio of OPA response of fermented samples (OPA_sample_) relative to that of acid hydrolysates (OPA_max_), as follows: PI = OPA_sample_/OPA_max,_ expressed as a percentage.

The quantified FAA and the overall NH_2_ group values were used to calculate the content in peptides by difference between the total NH_2_ values and the FAA values, converted in NH_2_ content.

### Statistical analyses.

Analyses of variance (ANOVA) were performed to determine whether the acidification and growth parameters, the carbohydrate and volatile-compound contents, differed according to the mode of culture used (mono- or coculture), using the function aov of R (R version 3.5.1; RStudio, Inc.). Means were compared using the Tukey *post hoc* test from the package car of R (*P* value < 0.05). ANOVA for the acidification parameters and carbohydrate hydrolysis were made on the whole data set gathering the three monocultures of donor strains, the three monocultures of the receiver strains, and the nine cocultures. For volatiles, ANOVA were made on the ratio of each compound compared to the control (fold change) within four data subsets: Efa2412 mono- and cocultures, Lla2125 monoculture and cocultures, Lla244 mono- and cocultures, and the three receiver strains grown in CDM TRYP.

Principal-component analyses (PCA) and a hierarchical clustering on principal components (HCPC) were performed using the FactoExtra package of R.
